# A trivalent inactivated PDCoV-PEDV-TGEV vaccine confers protective efficacy in piglets coinfected with porcine rotavirus

**DOI:** 10.3389/fvets.2026.1879116

**Published:** 2026-07-02

**Authors:** Yinhe Zha, Xiaoyu Yu, Yu Duan, Wentao Zhang, Fang Wang, Wenhao Chen, Zhikun Zou, Yulong He, Jianhong Shu

**Affiliations:** 1 College of Life Sciences and Medicine, Zhejiang Sci-Tech University, Hangzhou, China; 2 Zhejiang Hom-Sun Biotechnology Co. Ltd., Shaoxing, China

**Keywords:** coinfection, immune protection, neutralizing antibody, porcine coronavirus, porcine rotavirus, trivalent inactivated vaccine

## Abstract

Porcine epidemic diarrhea virus (PEDV), porcine deltacoronavirus (PDCoV), and transmissible gastroenteritis virus (TGEV) are the primary enteric coronaviruses responsible for viral diarrhea in piglets. These pathogens frequently co-circulate with porcine rotavirus (PoRV) under field conditions, leading to increased disease severity and complicating prevention efforts. Although multivalent vaccines targeting these three coronaviruses have been developed, their protective efficacy in the presence of PoRV coinfection remains largely unknown. Based on our previously developed trivalent inactivated PDCoV-PEDV-TGEV vaccine, the present study evaluated its immunoprotective efficacy in piglets naturally infected with PoRV. The results showed that, despite the background of persistent PoRV infection, the trivalent vaccine induced detectable neutralizing antibodies against PEDV, PDCoV, and TGEV (titers≥1:64). Furthermore, vaccination significantly reduced fecal viral shedding after challenge, shortened the duration of diarrhea, and alleviated intestinal pathological damage. Immunofluorescence assays confirmed that antigen deposition of the target coronaviruses in the intestines of vaccinated piglets was markedly reduced. However, vaccinated piglets continued to shed PoRV, indicating that the vaccine did not confer sterilizing immunity but rather reduced clinical severity. This study provides the experimental evidence that the trivalent inactivated vaccine confers effective protection against the three major porcine enteric coronaviruses even under complex clinical conditions involving PoRV coinfection. These findings offer important insights for developing immunization strategies to control multi-pathogen infections in swine production.

## 1 Introduction

Porcine epidemic diarrhea virus (PEDV), porcine deltacoronavirus (PDCoV), and transmissible gastroenteritis virus (TGEV) are three major enteric pathogens that collectively inflict substantial economic losses on the global swine industry. These pathogens primarily infect newborn and nursing piglets, leading to clinical signs such as severe watery diarrhea, vomiting, dehydration, and elevated mortality rates. PEDV, a member of the genus *Alphacoronavirus*, spreads through fecal-oral transmission, aerosol exposure, and contaminated milk. Its pathogenesis involves severe villous atrophy and epithelial necrosis in the small intestine (1, 2). PDCoV belongs to the genus *Deltacoronavirus* and was first identified in fecal samples from pigs in Hong Kong in 2012 (3, 4). Like PEDV, PDCoV transmits primarily through the fecal-oral route and causes similar clinical signs, though its pathogenicity is generally considered milder (5). TGEV, also an *Alphacoronavirus*, targets villous epithelial cells in the small intestine, resulting in villus atrophy and malabsorptive diarrhea (6–8). Importantly, these three coronaviruses are frequently detected together in field settings, complicating differential diagnosis and disease control (9–11).

Porcine rotavirus (PoRV), a member of the family *Reoviridae*, is another ubiquitous pathogen causing diarrhea in piglets worldwide. PoRV damages intestinal epithelial cells, leading to malabsorption, and its clinical manifestations closely resemble those caused by coronaviruses (12). Coinfections not only exacerbate clinical symptoms but may also compromise vaccine efficacy through immune interference or synergistic pathogenic mechanisms (13, 14). Therefore, evaluating vaccine performance under multi-pathogen conditions that mirror real-world scenarios is critically important.

Currently, various vaccines targeting PEDV, PDCoV and TGEV have made substantial progress. Most commercial vaccines for controlling viral diarrhea in piglets primarily target PEDV and TGEV, with some formulated as bivalent or trivalent combinations including PoRV (15–19). However, their broad-spectrum protection remains suboptimal. Single-target vaccines cannot effectively control complex diarrhea caused by multiple pathogens, while administering multiple vaccines increases costs and labor requirements (15, 20). As swine production intensifies, a trivalent inactivated vaccine simultaneously targeting PEDV, PDCoV, and TGEV offers a promising approach to broaden pathogen coverage while reducing immunization frequency and cost (15, 20, 21).

In our previous study, we successfully developed a trivalent inactivated vaccine against PEDV, TGEV, and PDCoV. This vaccine induced high neutralizing antibody levels, effectively alleviated clinical symptoms, reduced viral shedding, and mitigated intestinal lesions in single-virus infection models (21). However, its protective efficacy under the more complex and clinically relevant condition of PoRV coinfection remained to be determined.

To address this knowledge gap, we selected piglets naturally infected with PoRV as experimental animals and evaluated the immunoprotective efficacy of our trivalent inactivated vaccine against PEDV, PDCoV, and TGEV challenge under persistent PoRV infection. This experimental design mimics the mixed infection scenarios frequently encountered in commercial swine herds, providing systematic evidence for assessing the field applicability of this trivalent vaccine and informing integrated approaches for controlling multi-pathogen enteric diseases.

## 2 Materials and methods

### 2.1 Cell lines, viral strains, and reagents

The ST, Vero, and LLC-PK1 cell lines were maintained in DMEM (Gibco, USA) containing 10% fetal bovine serum (FBS; Gibco, USA) and incubated at 37 °C with 5% CO_2_ in a humidified atmosphere. The TGEV SHXB (GenBank: KP202848), PEDV ZJ/15 (GenBank: KX550281.1), and PDCoV LYG/14 (GenBank: KU665558.1) strains were isolated from diseased piglets in China. For virus propagation, TGEV, PEDV, and PDCoV were inoculated into ST, Vero, and LLC-PK1 cells, respectively, using maintenance medium containing 10 μg/mL TPCK-treated trypsin (Gibco, USA). Infectious titers were measured as TCID50 values and calculated using the Reed-Muench approach.

### 2.2 Preparation of a trivalent inactivated vaccine against TGEV, PEDV, and PDCoV

Virus stocks were amplified in their respective susceptible cell systems until titers reached 10^8.5^ TCID_50_/mL for TGEV and 10^8.0^ TCID_50_/mL for PEDV and PDCoV. Culture fluids were collected and clarified by centrifugation at 3,000 × g for 20 min at 4 °C, followed by filtration through 0.22 μm polyethersulfone (PES) membranes. To ensure rigorous formulation reproducibility and uniform batch payload delivery, individual viral components were quantitatively standardized back to these baseline pre-inactivation infectious titers prior to combining. Inactivation was achieved by adding *β*-propiolactone (BPL) to a final concentration of 0.05% and incubating at 4 °C for 24 h. Residual BPL was subsequently hydrolyzed at 37 °C for 2 h. Complete inactivation was confirmed by three blind passages in permissive cells, with no cytopathic effects or viral growth observed. The inactivated antigens were mixed at a volume ratio of 5:1:3 (PEDV: TGEV: PDCoV) and emulsified with M108L adjuvant (Hangzhou YiSiKang Pharmaceutical Technology, China) at a 9:1 (v/v) ratio. This 9:1 emulsification ratio was strictly designated in accordance with the manufacturer’s technical specifications for the proprietary M108L micro-emulsion system to limit the single actual injection volume to 2 mL while ensuring structural emulsion stability and safety for use in neonatal animal tissues. The final trivalent formulation was stored at 4 °C until use.

### 2.3 Immunization and challenge protocol in piglets

All animal procedures were approved by the Institutional Animal Care and Use Committee of Zhejiang Sci-Tech University (Approval No. Sci-Tech-SW-2024-008) and conducted in accordance with ARRIVE guidelines. A total of 33 3-day-old piglets acquired from a commercial farm were confirmed to be free of PEDV, PDCoV, and TGEV (no antigens or antibodies detected), but were positive for PoRV antigen. Infection was confirmed by RT-qPCR on fecal samples (Primers and TaqMan probes are listed in Table 1). At 3 days of age prior to their first vaccine dose, the baseline PoRV replication levels varied drastically among individual piglets, spanning from high viral loads (Ct ~ 22.17) to trace baseline metrics (Ct > 37.89). To mitigate baseline confounding variables, piglets were stratified according to initial Ct profiles to distribute baseline viral heterogeneity equally across all designated study blocks.

**Table 1:** qPCR primer and probe sequences.

Gene	Primer name	Primer sequence (5′ → 3′)
TGEV-N	TGEV-N-Probe	TCTTTCATTCTTCAACCCCATAACCCTCCA
	qTGEV-N-F	CCCGTGGTCGGAAGAGTAATAA
	qTGEV-N-R	GGGTACAAAGTCTCTCGGACATAAG
PEDV-N	PEDV-N-Probe	CTGTTGTTGCCATTGCCACGA
	qPEDV-N-F	GTCTGAAAAGCCAATCATTC
	qPEDV-N-R	TTGCCTCTGTTGTTACTC
PDCoV-N	PDCoV-N-Probe	CACACCAGTCGTTAAGCATGGCAAGCT
	qPDCoV-N-F	ATCGACCACATGGCTCCAA
	qPDCoV-N-R	CAGCTCTTGCCCATGTAGCTT
PoRV-NSP5	PoRV-N-Probe	TCGAATGCAGTTAAGACARAYGCAGACGCT
	qPoRV -N-F	TCCACTMACCAGYTTTTCGAT
	qPoRV-N-R	AWGGYCGTGATTGYGYYGAT

Piglets were randomly divided into three experimental groups. The Trivalent Inactivated Vaccine Group, consisting of 15 animals, received 2 mL of the trivalent inactivated vaccine subcutaneously at 3 days of age (Prime), with a booster administered on day 17 (1st Boost). Another 15 piglets, serving as Non-Vaccinated Challenge Control Group, were injected with 2 mL of sterile PBS via the same route. The remaining three animals were maintained as Unchallenged Mock Group. The detailed group design is shown in Table 2.

**Table 2:** Immunization schedule and group allocation of piglets.

Group name	Number	Vaccination dose	Vaccination schedule (days of age)	Terminal necropsy (days of age)
Trivalent inactivated vaccine group	15	2 mL	Day 3 (Prime), Day 17 (1^st^ Boost)	Day 41
Non-vaccinated challenge control group	15	2 mL Sterile PBS	Day 3 (Prime), Day 17 (1^st^ Boost)	Day 41
Unchallenged mock group	3	None	Unvaccinated	Day 41

On day 27 of age (10 days following the booster), the vaccination and challenge control groups were further subdivided into three subgroups (*n* = 5 per subgroup) and challenged orally with 10 mL of either PEDV ZJ/15 (10^6.5^ TCID_50_/mL), TGEV SHXB (10^6.0^ TCID_50_/mL) or PDCoV LYG/14 (10^6.5^ TCID_50_/mL). The PEDV inoculum was derived from standardized clean cell culture viral suspensions. In contrast, the TGEV and PDCoV inocula were prepared directly from early-passage virulent intestinal homogenates to preserve robust enteropathogenicity, which rapidly attenuates during prolonged cell culture isolation. To remove endogenous tissue factors and potential microbial debris, these raw tissue matrices underwent three freeze–thaw cycles, high-speed clarification at 12,000 g for 30 min at 4 °C, and final sequential processing through 0.22 μm filters. Healthy control animals received 10 mL of DMEM. To validate the experimental blinding, all study groups were housed in separate, identical high-containment isolation units within the same centralized building to achieve perfect environmental standardization while preventing cross-contamination. Clinical signs were monitored daily for 14 days post-challenge (DPC) by two independent board-certified veterinarians blinded to the vaccination assignments. Diarrhea severity was scored blindly using the following criteria: 0, normal formed feces; 1, soft/pasty feces retaining shape; 2, semi-liquid feces without defined shape; and 3, watery diarrhea. A fecal score of 2 or higher indicated diarrhea in the animals. Daily rectal swab samples were collected for viral shedding assessment. The experimental design is outlined in Table 3.

**Table 3:** Challenge study design for 27-day-old piglets.

Group name	Number	Challenge day	Virus strain inoculum	Inoculum dose	Necropsy day
Trivalent inactivated vaccine group	5	Day 27	PEDV ZJ/15 (Cell-derived)	10 mL × 10^6.5^ TCID_50_/mL/pig	Day 41
Non-vaccinated challenge control group	5	Day 27			Day 41
Trivalent inactivated vaccine group	5	Day 27	TGEV SHXB (Tissue-derived)	10 mL × 10^6.0^ TCID_50_/pig	Day 41
Non-vaccinated challenge control group	5	Day 27			Day 41
Trivalent inactivated vaccine group	5	Day 27	PDCoV LYG/14 (Tissue-derived)	10 mL × 10^6.5^ TCID_50_/pig	Day 41
Non-vaccinated challenge control group	5	Day 27			Day 41
Unchallenged mock group	3	Day 27	Sterile DMEM Medium	10 mL	Day 41

Serum samples for neutralizing antibody assay were obtained from blood collected at 27 days of age (10 days following booster vaccination). After centrifugation at 3,000 × g for 10 min, the serum was heat-inactivated at 56 °C for 30 min and maintained at −80 °C prior to analysis. To eliminate serum-induced cytotoxicity on specific cell lines while maximizing protocol uniformity, all serum samples were tested at a standardized starting baseline dilution of 1:4. Sera were two-fold serially diluted and mixed with an equal volume of virus suspension containing 100 TCID_50_ of PEDV, TGEV, or PDCoV. The virus strains used in this assay were 100% homologous to the exact strains integrated into the trivalent formulation. After incubation at 37 °C for 1 h, the mixtures were transferred to 96-well plates seeded with confluent Vero (for PEDV), ST (for TGEV), or LLC-PK1 (for PDCoV) cells. Cultures were maintained in medium supplemented with TPCK-treated trypsin at appropriate concentrations. Cytopathic changes were monitored daily for up to 72 h. Neutralizing titers were expressed as the reciprocal of the highest serum dilution that prevented CPE in 50% of wells, determined according to the Reed-Muench method.

**Figure 1:**
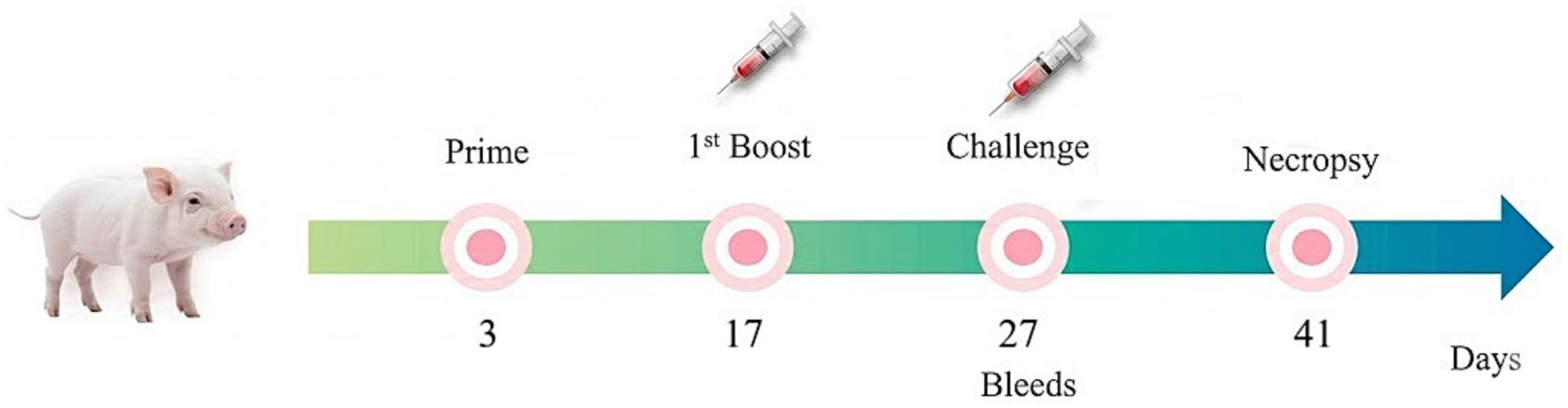
Trivalent vaccine immunization and challenge experimental timeline. The linear schematic outlines the chronological workflow: baseline endemic PoRV screening and primary immunization (Prime) at day 3 of age, booster injection (1st Boost) at day 17, pre-challenge serum sampling and subsequent oral virulent coronavirus challenge (PEDV, TGEV, or PDCoV) at day 27, followed by a 14-day post-challenge clinical monitoring window and final terminal sacrifice (Sacrifice) at day 41 of age.

The comprehensive operational procedure, including baseline screening, primary and booster immunizations, blood sampling, viral challenge, and the clinical tracking matrix, is schematically outlined in Figure 1.

### 2.4 Anesthesia and euthanasia of piglets

Zoletil 50 (tiletamine–zolazepam) was reconstituted according to the manufacturer’s instructions and administered via cervical intramuscular injection to each piglet at a volume of 1 mL per piglet (equivalent to approximately 5–10 mg/kg, adjusted according to body weight if necessary). After adequate anesthesia was confirmed, the piglets were euthanized by exsanguination through the left forelimb vessels. All procedures were conducted in accordance with ARRIVE guidelines. Humane endpoints were actively monitored every 6 h post-challenge. Piglets demonstrating severe clinical collapse, persistent recumbency with an inability to right themselves, unresponsive vomiting, or rapid dehydration exceeding 10% of total body weight were w euthanized humanely to restrict pain and distress.

### 2.5 Sample and tissue collection

Rectal swabs were collected during vaccination and daily after challenge, then immersed in 1 mL of sterile PBS. The samples were thoroughly vortexed and centrifuged at 12,000 × g for 10 min, and the resulting supernatants were preserved at −80 °C until RT-qPCR analysis. All piglets were euthanized at 14 days post-challenge (41 days of age), and intestinal tissues (duodenum, jejunum, ileum, cecum, colon, and rectum) were collected for viral load quantification. Additionally, sections of the ileum were fixed in 4% paraformaldehyde for subsequent histological, histomorphometric and immunofluorescence studies.

Total RNA was isolated from fecal and intestinal samples using standard methods, followed by reverse transcription with a Vazyme cDNA synthesis kit (Vazyme Biotech, China). Primers and TaqMan probes of PEDV, TGEV, PDCoV and PoRV were designed using Primer Express 3.0 (Applied Biosystems, USA) and are listed in Table 1. qPCR was performed in 20 μL volumes with AceQ qPCR Probe Master Mix (Vazyme Biotech, China).

### 2.6 Histopathology and indirect immunofluorescence

Ileal samples were fixed, processed through graded dehydration, embedded in paraffin, and sectioned at 5 μm. Hematoxylin–eosin staining was performed for microscopic examination. For indirect immunofluorescence, sections underwent deparaffinization, permeabilization with 0.1% Triton X-100, and blocking in 5% BSA. Slides were incubated overnight at 4 °C with specific mouse monoclonal antibodies against the N proteins of PEDV, TGEV, or PDCoV at a 1:200 dilution (monoclonal antibodies were prepared by our laboratory). Following washing steps, FITC-conjugated goat anti-pig IgG (Beyotime Biotechnology, China; 1:500) was applied for 1 h at 37 °C. Nuclear staining was carried out using DAPI, and fluorescence signals were visualized under a fluorescence microscope.

### 2.7 Statistical analysis

Statistical analyses were performed using GraphPad Prism 9.5 (GraphPad Software, USA). Neutralizing antibody titers and log_10_-transformed viral loads were analyzed by two-way ANOVA followed by Tukey’s multiple comparison post-hoc test. Daily ordinal non-parametric fecal diarrhea scores were evaluated statistically using the Kruskal-Wallis test followed by Dunn’s multiple comparison post-test. Data are presented as the mean ± standard error of the mean (SEM) or mean ± standard deviation (SD) where explicitly noted. Differences were considered statistically significant at *p* < 0.05 or *p* < 0.01.

## 3 Results

### 3.1 Confirmation of PoRV infection status in experimental piglets

Prior to the experiment, pre-screening for porcine rotavirus (PoRV) antigen confirmed that all 33 piglets were positive for PoRV; therefore, all challenge studies were performed under conditions of natural pre-existing infection. Throughout the experimental period, PoRV infection was detected at 3, 17, 27, and 41 days of age. The individual piglets exhibited fluctuating viral shedding patterns over time (Table 4). True sterilizing viral clearance was not achieved across all individuals, which represents an intrinsic characteristic of field multi-pathogen settings. However, a noticeable downward trend in replication was documented in several subjects; for instance, in the TGEV-vaccinated subgroup, the mean group Ct value shifted from 32.43 ± 1.82(SD) at day 3 to 32.35 ± 5.92 (SD) at day 41, with individual piglet Vac-10 successfully suppressing shedding down to a borderline negative baseline threshold (Ct = 39.10) at terminal necropsy (Table 4).

**Table 4:** Detection of endemic PoRV shedding (Ct values) in individual piglets during the vaccine immunization and challenge phases via RT-qPCR.

Virus strain inoculum	Group name	Piglet ID	Day 3 (Pre-Prime)	Day 17 (Pre-Boost)	Day 27 (Pre-Challenge)	Day 41 (Necropsy)
PEDV ZJ/15 (Cell-derived)	Trivalent Inactivated Vaccine Group	Vac-1	37.02	36.89	24.18	20.17
		Vac-2	33.23	33.35	24.10	25.34
		Vac-3	31.91	35.75	24.00	21.00
		Vac-4	31.38	35.84	27.67	27.60
		Vac-5	33.34	37.43	36.24	38.34
	Non-Vaccinated Challenge Control Group	Con-1	30.72	36.21	36.57	23.13
		Con-2	32.28	31.89	29.13	31.69
		Con-3	29.94	24.04	22.78	20.85
		Con-4	32.85	37.33	35.48	35.07
		Con-5	37.31	37.79	37.79	35.52
TGEV SHXB (Tissue-derived)	Trivalent Inactivated Vaccine Group	Vac-6	34.82	32.75	33.43	31.01
		Vac-7	32.34	36.10	39.10	35.90
		Vac-8	33.12	21.49	30.61	32.44
		Vac-9	30.16	30.65	23.81	23.31
		Vac-10	31.75	38.89	39.67	39.10
	Non-Vaccinated Challenge Control Group	Con-6	37.03	37.57	37.50	37.78
		Con-7	34.43	36.84	39.12	36.53
		Con-8	29.47	36.63	39.00	38.11
		Con-9	34.47	39.55	33.30	33.84
		Con-10	29.78	37.17	36.14	31.90
PDCoV LYG/14 (Tissue-derived)	Trivalent Inactivated Vaccine Group	Vac-11	28.46	29.32	33.52	31.35
		Vac-12	30.39	27.72	23.75	19.06
		Vac-13	32.93	36.81	36.61	34.65
		Vac-14	35.34	23.60	28.22	34.62
		Vac-15	35.17	29.94	20.13	22.41
	Non-Vaccinated Challenge Control Group	Con-11	30.66	25.59	25.12	23.32
		Con-12	33.18	38.87	39.86	37.29
		Con-13	34.85	35.20	39.22	37.31
		Con-14	22.17	18.45	30.78	31.26
		Con-15	37.89	34.51	28.83	23.12
Sterile DMEM medium	Unchallenged Mock Group	Moc-1	38.52	33.45	37.41	35.01
		Moc-2	31.10	21.02	26.25	34.26
		Moc-3	38.20	33.49	24.82	21.67

Individual piglet tracks are detailed horizontally across sequential chronological timepoints to display individual biological fluctuations. Higher Ct values signify lower viral replication; values ≥ 40.0 are considered negative.

### 3.2 Verification of viral inactivation

**Figure 2:**
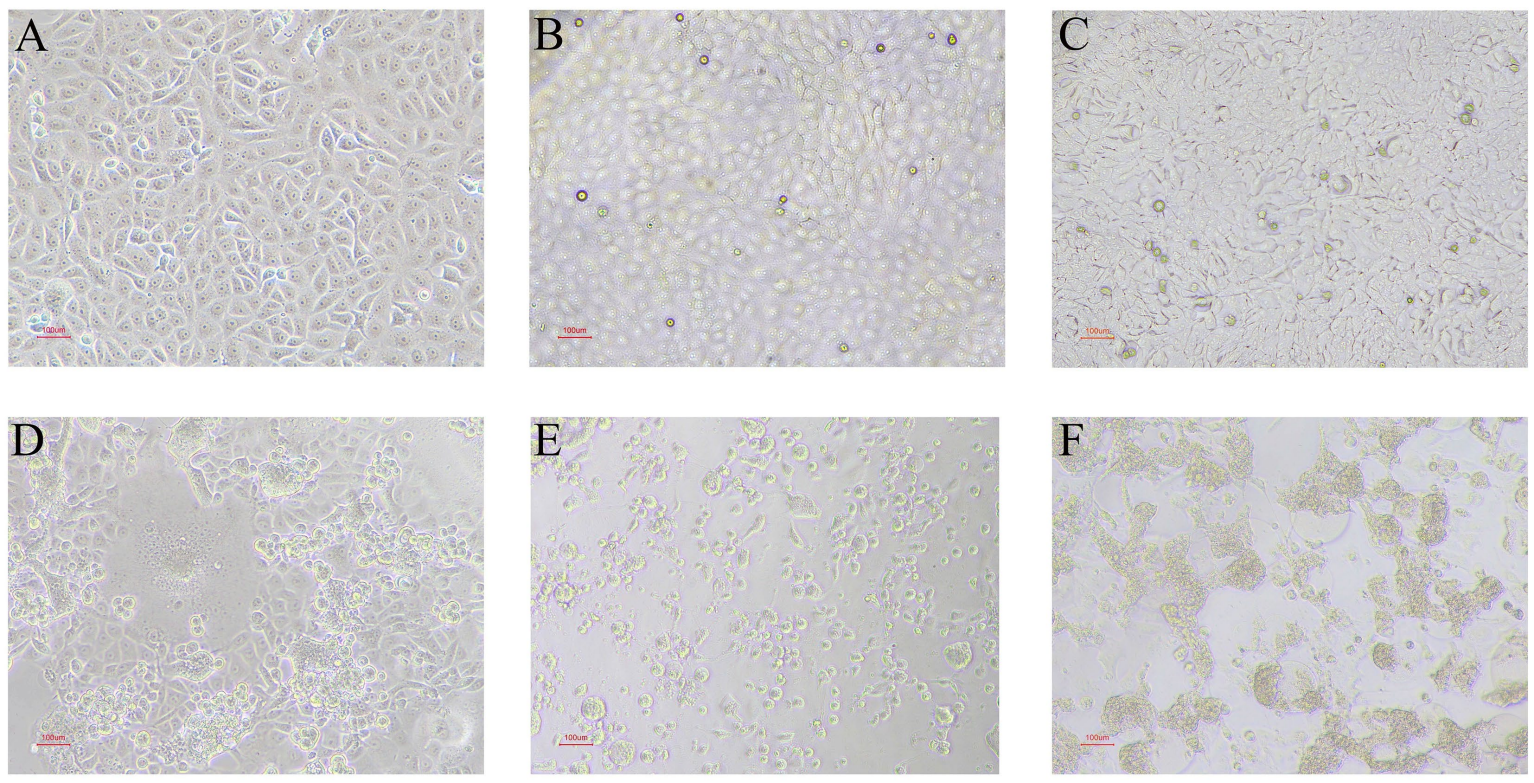
Cellular morphology before and after inoculation with TGEV, PEDV, and PDCoV. **(A)** Normal Vero cells. **(B)** Normal LLC-PK1 cells. **(C)** Normal ST cells. **(D)** Cytopathic effect (CPE) in Vero cells at 24 h post-inoculation (hpi) with PEDV. **(E)** CPE in LLC-PK1 cells at 24 hpi with PDCoV. **(F)** CPE in ST cells at 24 hpi with TGEV.

The three viral strains (TGEV, PEDV, and PDCoV) used for vaccine production were inactivated with BPL and subsequently subjected to three blind passages in ST, Vero, and LLC-PK1 cells, respectively. No CPE was observed in any of the inoculated cultures, while the negative control cells maintained normal morphology. The representative morphological characteristics of cells before and after inoculation with TGEV, PEDV, and PDCoV are presented in Figure 2, confirming complete viral inactivation.

### 3.3 Neutralizing antibody responses prior to challenge

**Figure 3:**
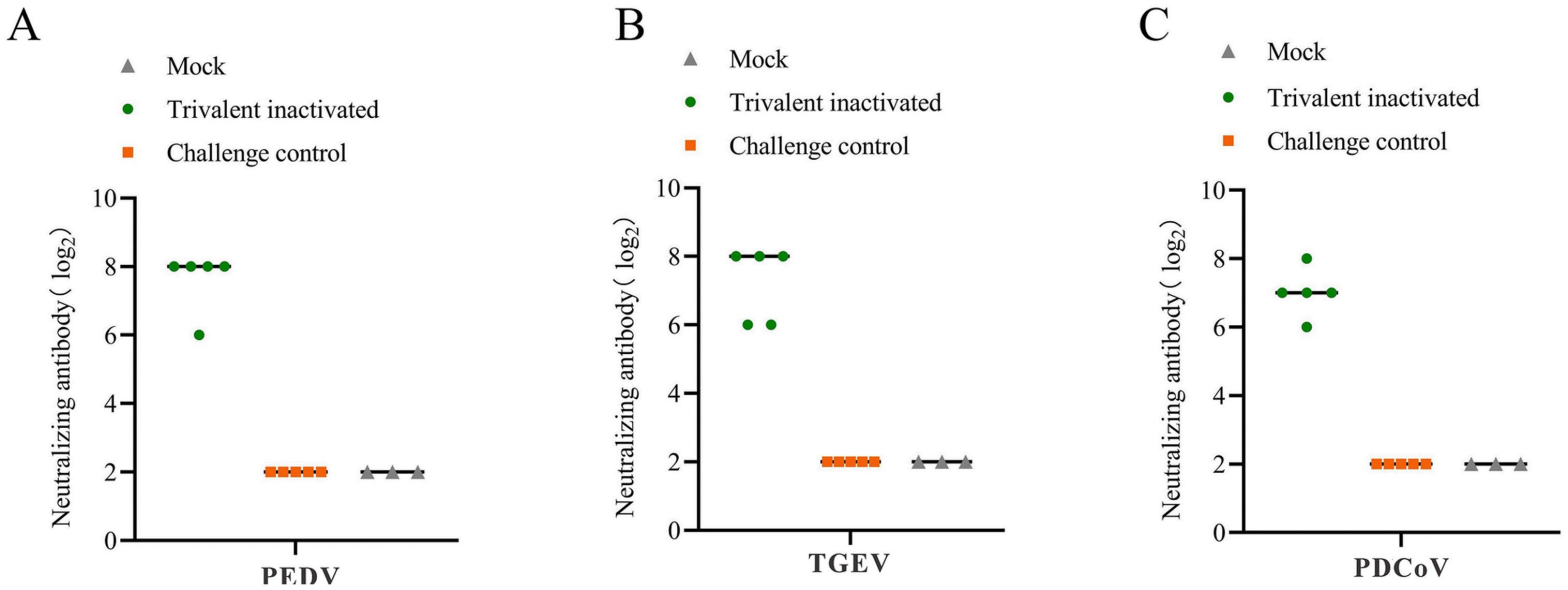
Serum neutralizing antibody titers in piglets before challenge(21 days post-vaccination). **(A)** Neutralizing antibody titers against PEDV. **(B)** Neutralizing antibody titers against TGEV. **(C)** Neutralizing antibody titers against PDCoV. Each symbol represents an individual piglet; horizontal bars indicate group means.

Serum samples collected at 27 days of age (10 days post-booster) were evaluated for neutralizing activity against PEDV, TGEV, and PDCoV. Piglets receiving the trivalent vaccine generated strong antibody responses to all three viruses. For PEDV, four of five animals (80%) exhibited titers of 1:256, while the remaining piglet reached 1:64 (Figure 3A). In the TGEV subgroup, three of five (60%) showed titers of 1:256 and the others maintained titers of 1:64 (Figure 3B). Regarding PDCoV, titers of 1:128 were detected in 80% (4/5) of piglets, with the lowest value recorded at 1:64 (Figure 3C). In contrast, sera from control animals remained below the detectable threshold. Collectively, these findings demonstrate that the trivalent inactivated formulation elicited measurable humoral immunity prior to challenge, despite the background of PoRV infection.

### 3.4 Clinical signs and diarrhea scoring following challenge

Animals were observed daily for 14 days following oral inoculation, and diarrhea severity was recorded. Due to pre-existing PoRV infection, diarrhea occurred in every challenged group (5/5, 100%). However, disease progression differed between groups. Kruskal-Wallis analysis of the daily diarrhea scores revealed that vaccinated piglets displayed significantly milder clinical manifestations, shorter overall diarrhea duration, and significantly accelerated clinical recovery rates (*p* < 0.05) compared to non-vaccinated controls. Conversely, symptoms in challenge controls were more pronounced and persisted longer. Individual daily scores are summarized in Tables 5–7.

**Table 5:** Diarrhea scores of pigs challenged with PEDV.

DPC	Trivalent inactivated vaccine group			Non-vaccinated challenge control group		
	Fecal consistency			Fecal consistency		
	Normal	Mild diarrhea	Watery diarrhea	Normal	Mild diarrhea	Watery diarrhea
1	0/5	2/5	3/5	2/5	0/5	3/5
2	0/5	2/5	3/5	1/5	1/5	3/5
3	0/5	0/5	5/5	2/5	0/5	3/5
4	1/5	2/5	2/5	0/5	5/5	0/5
5	0/5	5/5	0/5	5/5	0/5	0/5
6	0/5	5/5	0/5	1/5	4/5	0/5
7	3/5	2/5	0/5	1/5	4/5	0/5
8	3/5	2/5	0/5	2/5	3/5	0/5
9	4/5	1/5	0/5	1/5	4/5	0/5
10	5/5	0/5	0/5	5/5	0/5	0/5
11	5/5	0/5	0/5	5/5	0/5	0/5
12	5/5	0/5	0/5	5/5	0/5	0/5
13	5/5	0/5	0/5	5/5	0/5	0/5
14	5/5	0/5	0/5	5/5	0/5	0/5

**Table 6:** Diarrhea scores of pigs challenged with TGEV.

DPC	Trivalent inactivated vaccine group			Non-vaccinated challenge control group		
	Fecal consistency			Fecal consistency		
	Normal	Mild diarrhea	Watery diarrhea	Normal	Mild diarrhea	Watery diarrhea
1	0/5	0/5	5/5	0/5	0/5	5/5
2	0/5	0/5	5/5	0/5	0/5	5/5
3	0/5	1/5	4/5	0/5	1/5	4/5
4	0/5	1/5	4/5	0/5	1/5	4/5
5	0/5	1/5	4/5	0/5	0/5	5/5
6	0/5	5/5	0/5	0/5	2/5	3/5
7	0/5	5/5	0/5	1/5	3/5	1/5
8	0/5	5/5	0/5	0/5	3/5	2/5
9	3/5	2/5	0/5	0/5	5/5	0/5
10	0/5	5/5	0/5	0/5	3/5	2/5
11	0/5	5/5	0/5	0/5	3/5	2/5
12	0/5	5/5	0/5	0/5	5/5	0/5
13	1/5	4/5	0/5	0/5	4/5	1/5
14	1/5	4/5	0/5	0/5	5/5	0/5

**Table 7:** Diarrhea scores of pigs challenged with PDCoV.

DPC	Trivalent inactivated vaccine group			Non-vaccinated challenge control group		
	Fecal consistency			Fecal consistency		
	Normal	Mild diarrhea	Watery diarrhea	Normal	Mild diarrhea	Watery diarrhea
1	0/5	2/5	3/5	0/5	0/5	5/5
2	0/5	2/5	3/5	0/5	0/5	5/5
3	0/5	2/5	3/5	0/5	0/5	5/5
4	0/5	5/5	0/5	0/5	0/5	5/5
5	0/5	5/5	0/5	0/5	1/5	4/5
6	1/5	4/5	0/5	0/5	5/5	0/5
7	0/5	5/5	0/5	0/5	5/5	0/5
8	2/5	3/5	0/5	1/5	4/5	0/5
9	3/5	2/5	0/5	1/5	4/5	0/5
10	5/5	0/5	0/5	3/5	2/5	0/5
11	5/5	0/5	0/5	4/5	1/5	0/5
12	5/5	0/5	0/5	4/5	1/5	0/5
13	5/5	0/5	0/5	4/5	1/5	0/5
14	5/5	0/5	0/5	0/5	5/5	0/5

### 3.5 Viral shedding kinetics after challenge

**Figure 4:**
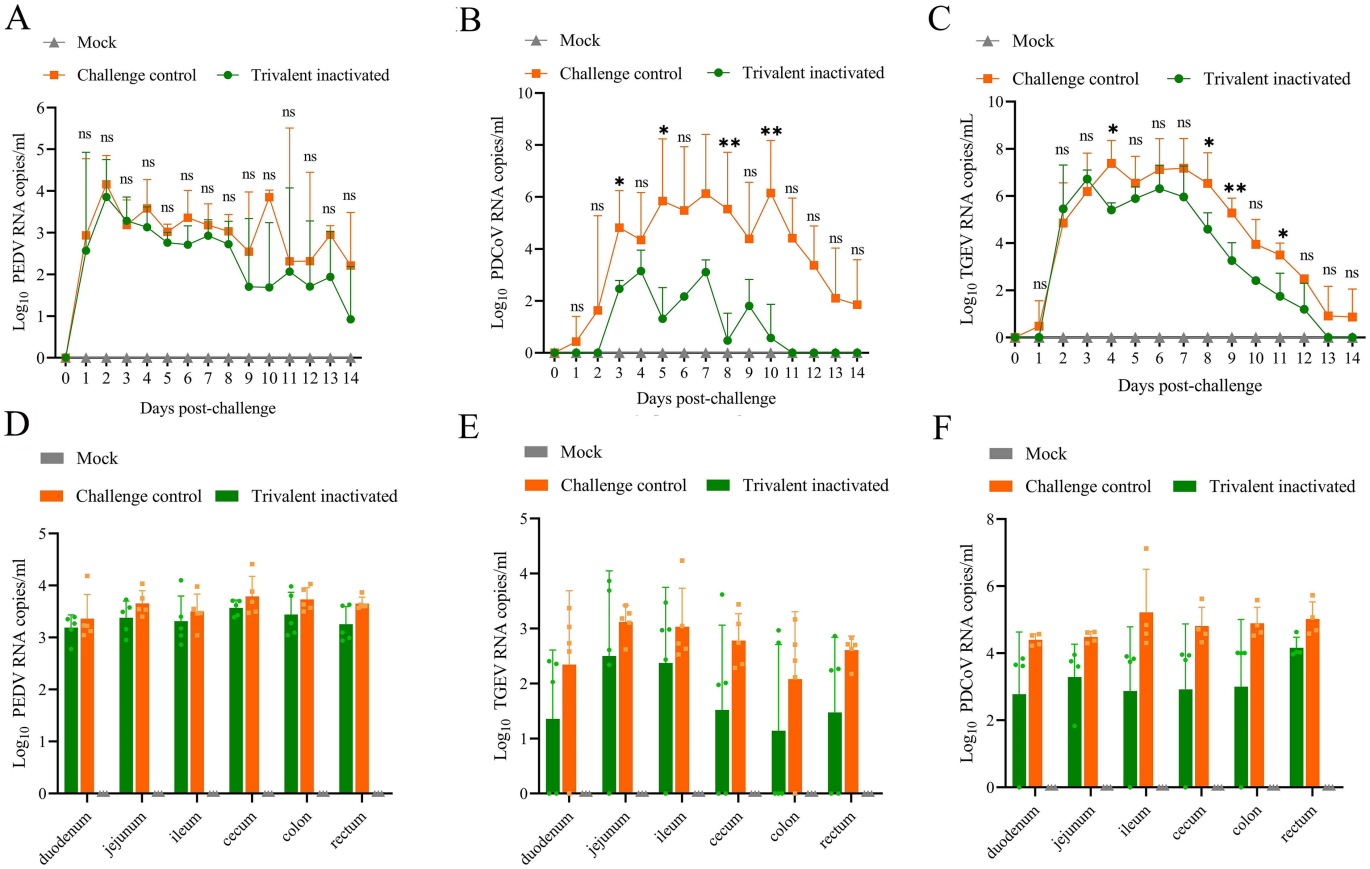
Fecal viral shedding dynamics and intestinal viral load post-challenge. **(A)** PEDV-challenged group. **(B)** PDCoV-challenged group. **(C)** TGEV-challenged group. **(D)** Viral load of PEDV in various intestinal tissues. **(E)** Viral load of PDCoV in various intestinal tissues. **(F)** Viral load of TGEV in various intestinal tissues. *p < 0.05, **p < 0.01 compared to challenge control group.

Fecal samples collected daily over 14 days were analyzed for viral RNA by RT-qPCR (Figures 4A–C). Under PoRV coinfection conditions, distinct shedding patterns were observed among the three coronaviruses. In non-vaccinated controls, PEDV RNA was detectable from 2 to approximately 11 DPC at consistently high levels. In contrast, vaccinated piglets maintained lower viral loads, with a noticeable decline after 8–9 DPC. PDCoV shedding in control animals persisted with peak levels between 6 and 8 DPC, whereas vaccinated piglets showed only brief early detection (around 2–6 DPC) followed by rapid reduction. A similar pattern was noted for TGEV: control piglets shed virus from 2 to roughly 11 DPC, reaching maximal levels at 4–7 DPC, while vaccination markedly shortened the shedding period and reduced RNA abundance, which approached minimal levels by 12–13 DPC.

At necropsy, viral RNA distribution was further assessed in multiple intestinal segments (Figures 4D–F). High viral burdens were present throughout both small and large intestines of challenge controls, including the duodenum, jejunum, ileum, cecum, colon, and rectum. In contrast, vaccinated animals showed substantially lower RNA levels across most regions, particularly within the jejunum and ileum. These results indicate that immunization effectively restricted intestinal replication and reduced fecal shedding of all three viruses under PoRV coinfection conditions.

### 3.6 Intestinal lesions and immunofluorescence detection

**Figure 5:**
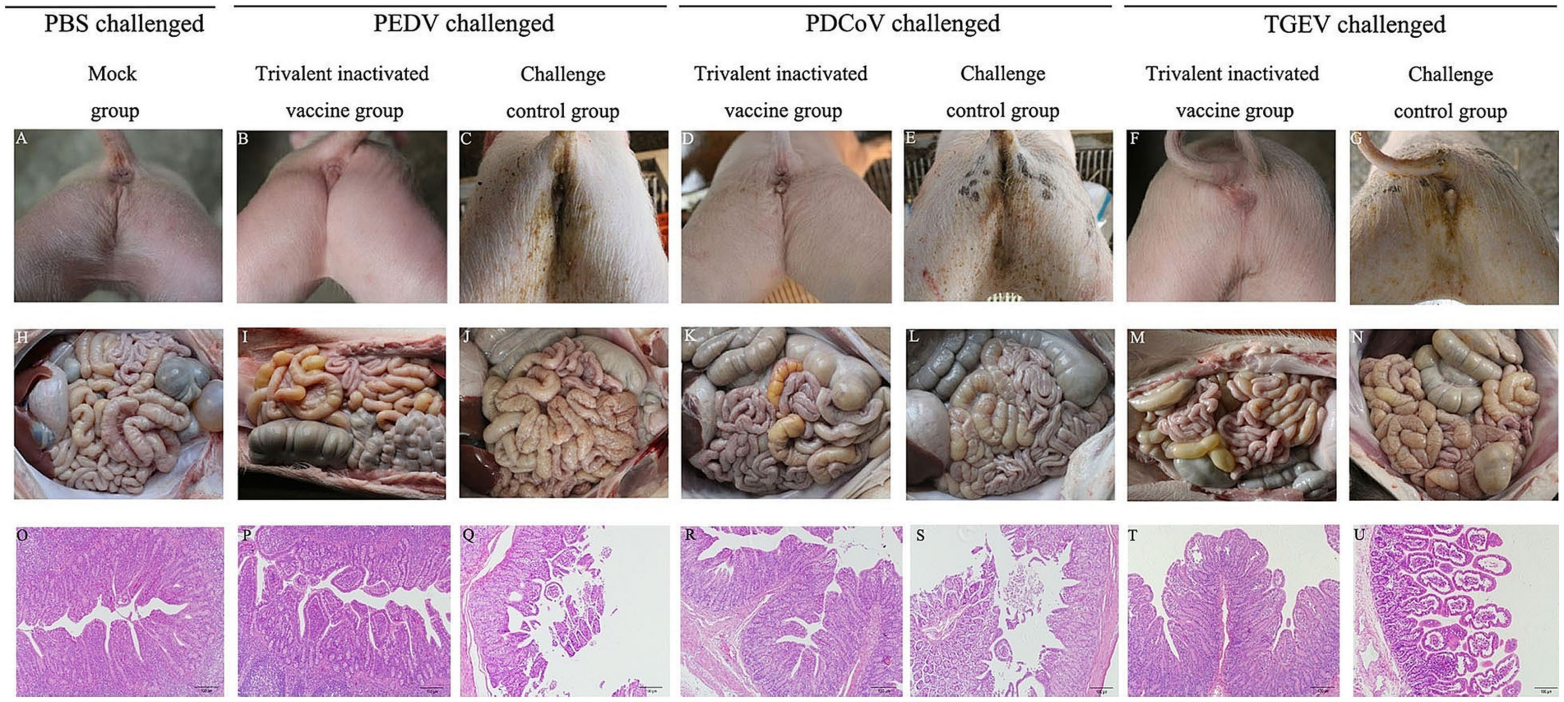
Clinical signs, gross intestinal lesions, and histopathological changes post-challenge. **(A–G)** Representative images depicting clinical manifestations. **(H–N)** Corresponding gross lesions of the intestinal tract. **(O–U)** Hematoxylin and eosin (H&E) staining of intestinal sections showing histopathological features. **(A,H,O)** Mock-challenged control group (PBS-inoculated), showing no clinical signs or macroscopic lesions. For each virus challenge: Vaccinated groups (clinical signs shown in **B,D,F**; gross lesions in **I,K,M**; H&E staining in **P,R,T**) exhibited mild symptoms and minor lesions, whereas challenge control groups (clinical signs in **C,E,G**; gross lesions in **J,L,N**; H&E staining in **Q,S,U**) developed severe symptoms and marked lesions. The viruses correspond as follows: PEDV **(B,C,I,J,P,Q)**, PDCoV **(D,E,K,L,R,S)**, and TGEV **(F,G,M,N,T,U)**.

Macroscopic examination revealed evident small intestinal damage in non-immunized challenged piglets. These animals exhibited intestinal dilation, thinning of the intestinal wall, increased transparency, and accumulation of fluid contents. Such alterations were notably milder in vaccinated piglets(Figures 5O–U).

**Figure 6:**
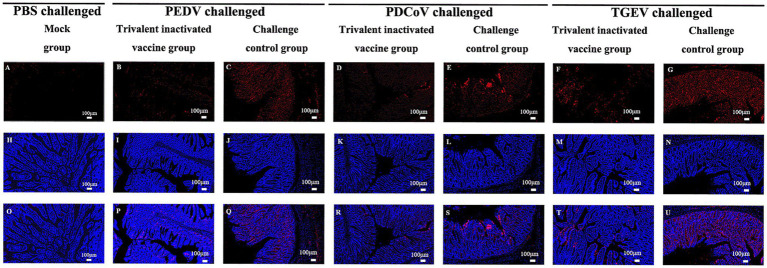
Immunofluorescence of ileal sections from challenged piglets (scale bar = 100 μm). Images show viral antigen (green, **A–G**), DAPI (blue, **H–N**), and merged channels **(O–U)**. **(A,H,O)** Mock control. For each virus (PEDV: **B,C,I,J,P,Q**; PDCoV: **D,E,K,L,R,S**; TGEV: **F,G,M,N,T,U**), the vaccinated groups **(P,R,T)** showed minimal antigen, while challenge controls **(Q,S,U)** exhibited abundant fluorescence, indicating vaccine protection during PoRV co-infection.

Indirect immunofluorescence staining demonstrated strong viral antigen signals within the mucosal epithelium of control animals. By comparison, only sparse and scattered fluorescence was detected in tissues from vaccinated piglets. Representative gross lesions are shown in Figures 5A–N, and reduced antigen distribution by IFA is presented in Figure 6.

## 4 Discussion

In modern swine production, enteric diseases rarely occur as single-pathogen events. This study evaluated a trivalent inactivated PDCoV-PEDV-TGEV vaccine under conditions of persistent porcine rotavirus (PoRV) infection, a background frequently encountered in commercial herds. The key findings are: (i) the vaccine induced neutralizing antibody titers ≥1:64 against all three coronaviruses despite PoRV coinfection; (ii) vaccinated piglets showed reduced fecal viral shedding, shorter diarrhea duration, and less pronounced intestinal damage compared to non-vaccinated controls; (iii) however, the vaccine did not confer sterilizing immunity, as vaccinated piglets continued to shed PoRV. These outcomes indicate that protective efficacy against coronaviruses was partially retained under coinfection conditions, but the vaccine’s limitations in blocking rotavirus shedding highlight the need for multivalent strategies that address all co-circulating pathogens.

For inactivated vaccines, antibody-mediated immunity remains the central protective mechanism. In this study, detectable neutralizing antibody titers against all three coronaviruses were observed in vaccinated animals, and these responses were not evidently compromised by ongoing PoRV infection. This is in line with previous observations linking circulating neutralizing antibodies with protection against PEDV (22, 23). Although viral coinfections have been reported to interfere with antigen presentation and cytokine signaling pathways (13, 24), the immune response generated in this model appeared sufficient to overcome such potential modulation. Protective effects may involve systemic IgG reaching the intestinal lumen through transudation, while mucosal immunity—particularly secretory IgA (sIgA)—is recognized as a critical mediator of viral neutralization at epithelial surfaces (25, 26).

Assessment of vaccine performance relied on clinical observation, virological quantification, and tissue-level analysis. Diarrhea severity was reduced and recovery occurred earlier in vaccinated piglets. Fecal viral RNA levels were consistently lower than those recorded in non-immunized controls, a pattern comparable to previous vaccination reports (21). After PDCoV challenge, the shortened shedding period in immunized animals suggests more efficient viral clearance. Microscopic evaluation further demonstrated preservation of villus morphology, accompanied by decreased viral antigen detection within enterocytes. Because PEDV, PDCoV, and TGEV preferentially infect mature intestinal epithelial cells, villus atrophy closely parallels disease severity and absorptive dysfunction (1, 6, 27, 28). Maintenance of villus structure therefore reflects meaningful protection at both structural and functional levels.

An additional strength of this work lies in the use of a complex, real-world coinfection model rather than a pristine experimental design. Rather than relying on specific pathogen-free or single-virus challenge systems, the model incorporated pre-existing rotavirus infection, better approximating field conditions. The persistence of vaccine efficacy under these circumstances suggests a degree of immunological stability and supports its application in herds where rotavirus circulation is endemic. Nevertheless, the use of intestinal homogenates for TGEV and PDCoV challenge (as opposed to cell-culture purified virus for PEDV) may have introduced additional inflammatory mediators, potentially confounding the comparison of challenge outcomes. This limitation should be addressed in future studies by using uniformly purified challenge materials.

A limitation of this study is the variation in baseline PoRV loads among individual piglets (Ct range 22.17–38.52 at 3 days of age). Although random group allocation ensured no significant differences between treatment arms, this biological variance may have influenced initial vaccine antigen processing. Future studies should consider screening for narrower baseline ranges or using statistical adjustment methods (e.g., ANCOVA) to account for this variability.

Furthermore, the vaccine did not prevent PoRV shedding (Table 4). This was expected because the trivalent vaccine targets only coronaviruses, not rotavirus. The persistent PoRV shedding underscores the importance of developing multivalent vaccines that include rotavirus antigens or implementing combination immunization strategies in the field.

Certain constraints should be considered when interpreting these findings. The experiments focused on direct immunization of piglets, whereas passive lactogenic immunity is widely acknowledged as the dominant protective mechanism in neonates. Colostrum- and milk-derived sIgA play pivotal roles in defense against PEDV and TGEV infection (25, 29, 30). In addition, the duration of follow-up was limited, preventing evaluation of long-term immune persistence. Cellular immune parameters were not systematically characterized, although T-cell responses contribute to affinity maturation and memory development (31). Interactions between PoRV and enteric coronaviruses at the level of epithelial receptors or innate antiviral signaling pathways, including interferon-mediated responses, remain to be clarified and merit further investigation in controlled *in vitro* systems (13, 32).

In conclusion, this trivalent inactivated PDCoV-PEDV-TGEV vaccine demonstrated partial protective capacity against three major enteric coronaviruses despite continuous PoRV exposure. The vaccine reduced clinical severity and accelerated viral clearance, but did not confer sterilizing immunity as vaccinated piglets continued to shed PoRV. Future optimization should focus on enhancing mucosal immunity to block shedding, incorporating rotavirus antigens into the multivalent formulation, and evaluating maternal antibody transfer.

## Data Availability

The original contributions presented in the study are included in the article/supplementary material, further inquiries can be directed to the corresponding author.
